# Synthesis, Characterization, and Study of In Vitro Cytotoxicity of ZnO-Fe3O4 Magnetic Composite Nanoparticles in Human Breast Cancer Cell Line (MDA-MB-231) and Mouse Fibroblast (NIH 3T3)

**DOI:** 10.1186/s11671-016-1734-9

**Published:** 2016-12-02

**Authors:** Gunjan Bisht, Sagar Rayamajhi, Biplab KC, Siddhi Nath Paudel, Deepak Karna, Bhupal G. Shrestha

**Affiliations:** 1 Department of Chemical Science and Engineering, Kathmandu University, Dhulikhel, Post Box 6250, Kathmandu, Nepal; 2 Department of Biotechnology, Kathmandu University, Dhulikhel, Nepal

**Keywords:** ZnO-Fe_3_O_4_ composite, ZnO nanoparticles, Magnetic nanoparticles, Ex situ conjugation, In vitro cytotoxicity, MDA-MB-231, NIH 3T3

## Abstract

**Abstract:**

Novel magnetic composite nanoparticles (MCPs) were successfully synthesized by ex situ conjugation of synthesized ZnO nanoparticles (ZnO NPs) and Fe_3_O_4_ NPs using trisodium citrate as linker with an aim to retain key properties of both NPs viz. inherent selectivity towards cancerous cell and superparamagnetic nature, respectively, on a single system. Successful characterization of synthesized nanoparticles was done by XRD, TEM, FTIR, and VSM analyses. VSM analysis showed similar magnetic profile of thus obtained MCPs as that of naked Fe_3_O_4_ NPs with reduction in saturation magnetization to 16.63 emu/g. Also, cell viability inferred from MTT assay showed that MCPs have no significant toxicity towards noncancerous NIH 3T3 cells but impart significant toxicity at similar concentration to breast cancer cell MDA-MB-231. The EC50 value of MCPs on MDA-MB-231 is less than that of naked ZnO NPs on MDA-MB-231, but its toxicity on NIH 3T3 was significantly reduced compared to ZnO NPs. Our hypothesis for this prominent difference in cytotoxicity imparted by MCPs is the synergy of selective cytotoxicity of ZnO nanoparticles via reactive oxygen species (ROS) and exhausting scavenging activity of cancerous cells, which further enhance the cytotoxicity of Fe_3_O_4_ NPs on cancer cells. This dramatic difference in cytotoxicity shown by the conjugation of magnetic Fe_3_O_4_ NPs with ZnO NPs should be further studied that might hold great promise for the development of selective and site-specific nanoparticles.

**Graphical abstract:**

Schematic representation of the conjugation, characterization and cytotoxicity analysis of Fe_3_O_4_-ZnO magnetic composite particles (MCPs).
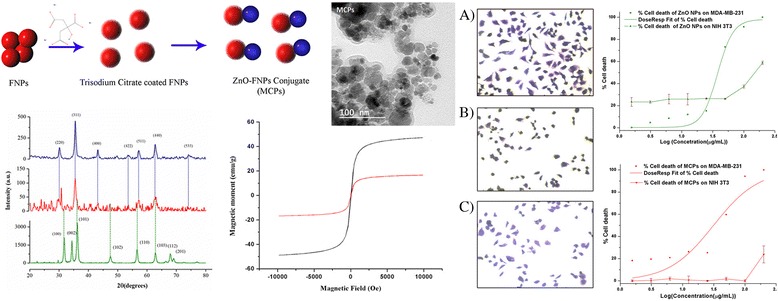

## Background

With novel approaches of cancer treatment being extensively researched in order to mitigate prime limitations of current approved therapeutics—nonspecificity and nonselective toxicity—development of targeted and selective cancer therapy has paved its way as a promising tool for next-generation cancer therapeutics by exploiting properties of specific binding like ligand-receptor binding, antigen-antibody mediated immune therapy etc. Targeted nanoparticles (NPs) also provide more therapeutic benefits besides specificity and specific localization like high payload, multidrug conjugation, easy tuning of release kinetics, selective localization, and bypass of multidrug resistance mechanism [1].

Ferrite magnetic NPs (Fe_3_O_4_ NPs) are of particular interest in biomedicine from hyperthermia application [2] to targeted therapy and drug delivery [3] because of its superparamagnetic behavior. Unlike strong ferromagnetic substances, they show linear dependence of magnetization with external magnetic field in the same direction with low maximum magnetization, a characteristic property of paramagnetism. But, the magnetic moment is four to ten times more than that of normal paramagnetic substances, hence considered as superparamagnetic [4]. This property allows Fe_3_O_4_ NPs to be used as effective vector for targeted drug therapy under the application of external field. However, it has high aggression and oxidation at physiological pH limiting its direct application which, nonetheless, can be overcome by biocompatible surface coating [3, 5]. But, at the same time, coating of Fe_3_O_4_ NPs with biocompatible nonmagnetic substance affects magnetization of the particle because of surface spin disorder, formation of dead magnetic zone, etc. which is strongly influenced by thickness and chemical composition of modifier [6]. Hence, tuning of modifier content for maintaining therapeutically significant magnetization value is very important for drug delivery application.

Another important nano-sized particle for cancer therapy is ZnO which has an inherent capacity to induce apoptosis in cancer cells while rendering very less toxic effect towards normal proliferating cells [7, 8]. Actual mechanism of this behavior is still not clear, but various arguments have been postulated; most supported being selective uptake of ZnO nanoparticles (ZnO NPs) [9] due to high expression of membrane anionic phospholipids on cancerous cells [10] imparting zinc-related ions disequilibrium inducing reactive oxygen species (ROS) [11, 12]. Nonetheless, this selectivity of ZnO NPs acts as promising tool for cancer treatment.

Since ZnO itself is biocompatible [13] and can act as a selective anticancer agent, conjugation of magnetic NPs with zinc oxide NPs could make novel therapeutically important compound. Recent research paper [14] has mentioned the synthesis of core-shelled ZnO-Fe_3_O_4_ NPs with molar ratios of Fe_3_O_4_ NPS to ZnO at 1:1, 1:10, and 1:20 which shows consecutive reduction of magnetization upon increasing ZnO proportion, being least reduced for 1:1. Although ample study on synthesis of ZnO-ferrite composite can be found as mentioned, there is still dearth on cytotoxicity study of such composite for possible cancer therapy. Hence, we tried to analyze the cytotoxic profile of this nanocomposite while still retaining therapeutically significant magnetization to be used in targeted localization.

## Methods

### Materials

Ferrous sulfate heptahydrate (FeSO_4_·7H_2_O), ferric chloride hexahydrate (FeCl_3_·6H_2_O), ammonium hydroxide (NH_4_OH), zinc acetate dihydrate (Zn(CH_3_COO)_2_·2H_2_O), sodium hydroxide (NaOH), trisodium citrate, methanol (CH_3_OH), and ethanol (C_2_H_5_OH) are of analytical grade purchased from Sigma-Aldrich and Amresco (USA).

### Synthesis of Magnetic Fe_3_O_4_ NPs

FeSO_4_·7H_2_0 (0.5 M) solution was mixed to FeCl_3_·6H_2_O (0.5 M) at 1:1 molar ratio in a conical flask placed on a magnetic stirring hot plate. NH_4_OH (1.5 M) was then added dropwise to iron solution maintained at 70 °C with vigorous magnetic agitation. Thus, precipitated black-colored particles were then filtered, washed with deionized water and ethanol, and then oven dried at 60 °C overnight. The dried particles were finally collected by magnetic separation.

### Synthesis of ZnO NPs

NaOH 0.25 M was added dropwise to 0.05 M zinc acetate maintained in a conical flask under vigorous mechanical agitation at room temperature; the cloudy viscous sediment thus obtained was filtered using vacuum filtration, dried overnight at 50 °C, and calcined at 200 °C for 2 h in a muffle furnace to obtain white fine powdered ZnO.

### Conjugation to Form MCPs

Ex situ conjugation approach as described by Shal and Jafari [14] was used to make the nanocomposite; 0.05 g of Fe_3_O_4_ NPs was dispersed in 0.05 M trisodium citrate solution prepared in 10 mL distilled water via shaking overnight at room temperature. Then, trisodium citrate-coated Fe_3_O_4_ NPs were washed with distilled water thrice and separated magnetically. Finally, 0.05 g of ZnO NPs was then added in a solution of trisodium citrate-coated Fe_3_O_4_ NPs resuspended on distilled water (pH 3.5 acidified by acetic acid) and stirred for 24 h at room temperature. Hence, formed magnetic composites were then separated magnetically, washed several times with distilled water, and dried at room temperature.

### Characterization

X-ray diffraction (XRD), transmission electron microscopy (TEM), and vibrating sample magnetometry (VSM) were carried out at IIT, Roorkee, India. XRD spectra were recorded at 0.154 nm wavelength (*λ*) of Cu-kα radiation using Rigaku-Geiger diffractometer with a range of 2*θ* from 5° to 80°. Phase identification and crystallographic planes were determined by comparing peak positions with reference JCPDS (Joint Committee on Powder Diffraction Standards) file. Particle size was computed using Scherrer’s equation: *D* = *Kλ*/*β*cos*θ*, where *K* = 0.9 is the shape factor, *β* is the full width at half maxima (FWHM) in radians, and *θ* is the Bragg’s angle [15]. For TEM analysis, required volume of sample was sonicated in acetone (1%, *w*/*v*) and applied over carbon-coated copper grid. TEM images were recorded at ×10K magnification and 0.2-μm scale over JEOL 1011 (Tokyo, Japan) at 80 kV. Similarly, Fourier transform infrared (FTIR) spectroscopy was performed on powder samples, and precursors using Shimadzu IR Prestige 21 FT-IR Spectrometer and corresponding spectrum was generated using IR-Solution software. VSM was performed using magnetometer (Princeton EG&G applied research model 155) with maximum current of 30 A, reading number 150, scan time 900 s at room temperature, and corresponding data were generated [15].

### Cell Culture

Cell lines MDA-MB-231 (human breast adenocarcinoma) and NIH 3T3 (murine fibroblast) were revived and maintained in Dulbecco’s modified Eagle’s medium (DMEM) (Life Technologies, USA) added up with 10% fetal bovine serum (FBS) (Sigma, Germany), 0.5% antibiotic solution (penicillin/streptomycin (Sigma, Germany) stabilized with glutamine), 0.5% antimycotic solution (Amphotericin “B” (Sigma, Germany)), and incubated at 37 °C by supplementing it with 5% CO_2_. Methyl thiazolyltetrazolium (MTT) dye (Amresco, USA) and Trypsin (Amresco, USA) were purchased. Stock solutions of ZnO NPs and magnetic composite nanoparticles (MCPs) were prepared in Dulbecco’s phosphate buffer saline (DPBS) (pH 7.4 ± 0.1). Prior to their use, the solutions were sonicated in water bath sonication for 1 h to prevent particle aggregation.

### Cell Viability Assay

MTT assay as mentioned by Adhikari et al. [16] was performed for cell viability assay. Highly proliferating log phase cells were harvested and seeded in 96-well plates in DMEM at density of 10^4^ cells per well. The seeded cells were left for attachment, and after 24 h, the media was replaced with fresh media supplemented with nanoparticles (ZnO NPs and MCPs) at concentration range from 200 μg/mL to eightfold serial dilution up to 1.56 μg/mL. After 24 h of treatment with NPs, cells were washed with DPBS twice and 10 μL of 5 mg/mL MTT dye was added. It was incubated for 4 h, and absorbance reading was taken at 570 nm with background subtraction of 630-nm band-pass filter. Percent cell viability was expressed as percent relative absorbance of sample respective to control.

### CV Staining

Cultured cells were seeded in 12-well plate at density of 80,000 cells per well and incubated for 24 h. These cells were treated with ZnO NPs and MCPs at near EC50 concentration of 40 and 30 μg/mL, respectively, and left for 24 h. Nonadherent cells were washed off using DPBS, and remaining cells were fixed with 4% paraformaldehyde for 30 min. Then, 0.05% of crystal violet solution in 20% ethanol was added and left to stain for 30 min. Finally, excess stain was washed off using distilled water and observed on phase contrast microscope at ×10 magnification.

### Computation and Statistical Analysis

Cytotoxicity was assessed by measuring cell viability as a function of relative absorbance, with respect to control (untreated cells), of dissolved formazan produced by the conversion of MTT dye by active mitochondrial dehydrogenase enzymes present in viable cells [17]. Dose-response was plotted under inbuilt logistic function on Origin 8 given by Eq. (1) as percent cell death versus logarithmic concentration. EC50 (measure for Potency), hill slope (*P*), and area under the curve (AUC) were assessed for cytotoxicity profile. Statistical analysis including ANOVA was done using inbuilt add-in of Microsoft Excel 2010.

1 $$ y=A1+\frac{A2-A1}{1+{10}^{\left( \log X0-X\right)p}} $$

where

A1 = bottom asymptote = 0 (fixed)

A2 = top asymptote = 100 (fixed)

Log*X*_0_ = mid-value of curve

*P* = slope factor (hill slope)

## Results and Discussion

### Characterization

XRD and TEM were performed for the analysis of size of synthesized particle and study of its morphological characteristics, respectively. FTIR was used to verify the conjugation of NPs on composite particle and to assess its purity. Magnetic properties of particles were studied by VSM, to verify whether as-synthesized MCPs retained magnetic property of Fe_3_O_4_ NPs.

### XRD and TEM Analysis

**Fig. 1:**
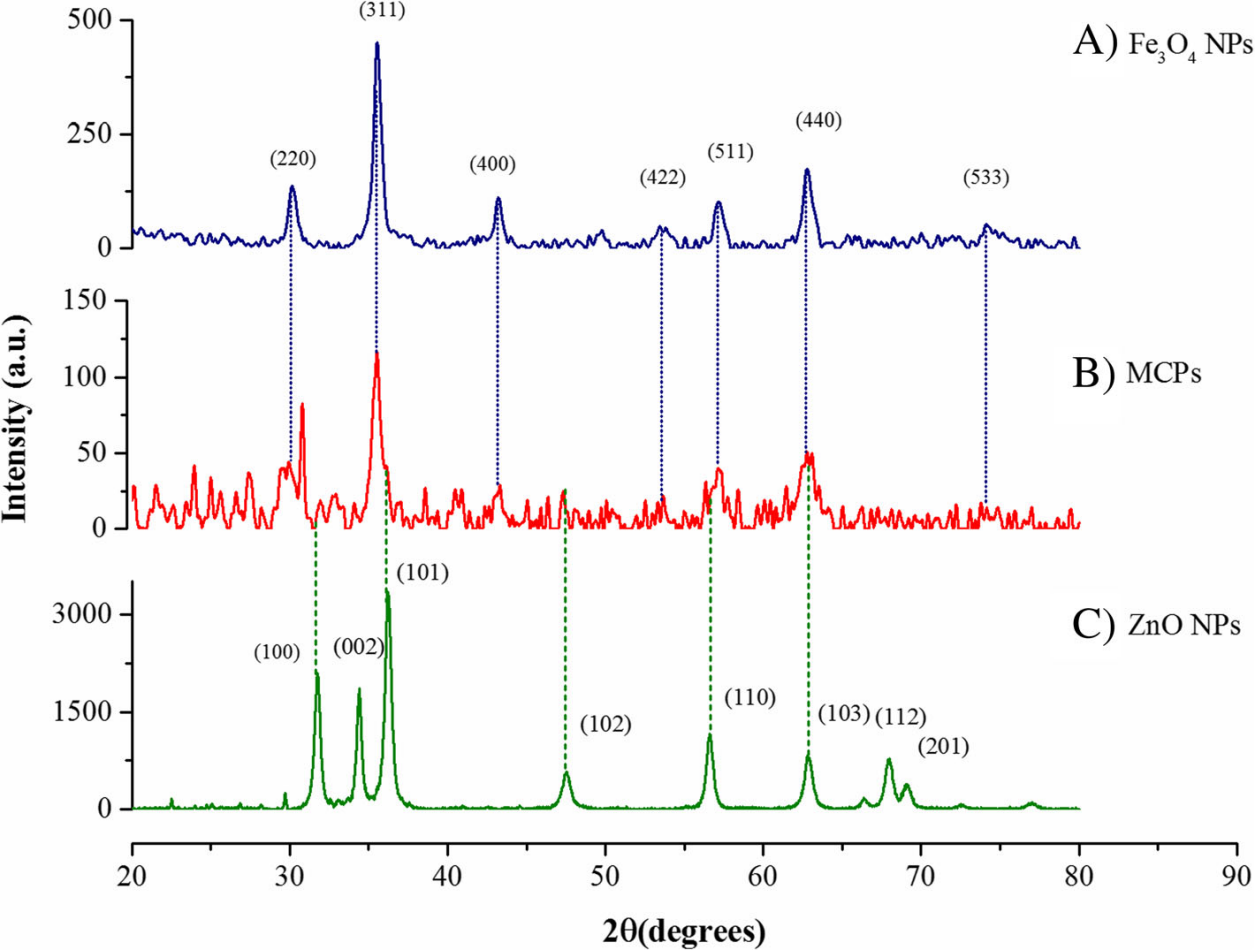
X-ray diffraction patterns of *A* Fe_3_O_4_, *B* magnetic composite NPs (MCPs), and *C* ZnO NPs. *Dotted lines* are used to correlate individual peaks of as-synthesized Fe_3_O_4_ and ZnO NPs with magnetic composite nanoparticles while values corresponding to miller indices (hkl values) are labeled for peaks between 2*θ* values of 20°–80°

X-ray diffraction pattern of synthesized particle is shown in Fig. 1. ZnO NPs show sharp diffraction peaks corresponding to hkl values of (100), (002), (101), and (110) at 2*θ* values of 31.765, 34.391, 36.195, and 56.606, respectively, suggesting its crystalline nature. Similarly, Fe_3_O_4_ NPs show diffraction peaks corresponding to hkl values of (220), (311), (422), and (553) at 2*θ* values of 30.07, 35.54, 43.14, and 62.78, respectively. Average particle size was obtained as 18.67 ± 2.2 nm for ZnO NPs and 14.56 ± 1.53 nm for Fe_3_O_4_ using Scherrer’s equation. Corresponding hkl values obtained from PowderX software indicate crystalline planes of polygonal Wurtzite structure of ZnO and inverse spinel structure of magnetite. Five distinct peaks were observed in the XRD of MCPs which can be correlated with individual peaks in singly constituted ZnO NPs and Fe_3_O_4_ NPs. Since characteristic XRD peaks in individual particles can be correlated with standard JCPDS file, with no apparent shift in their position, it suggests that a heterostructure is formed comprising of both the particles such that the MCP is crystalline in nature with no alloying.

**Fig. 2:**
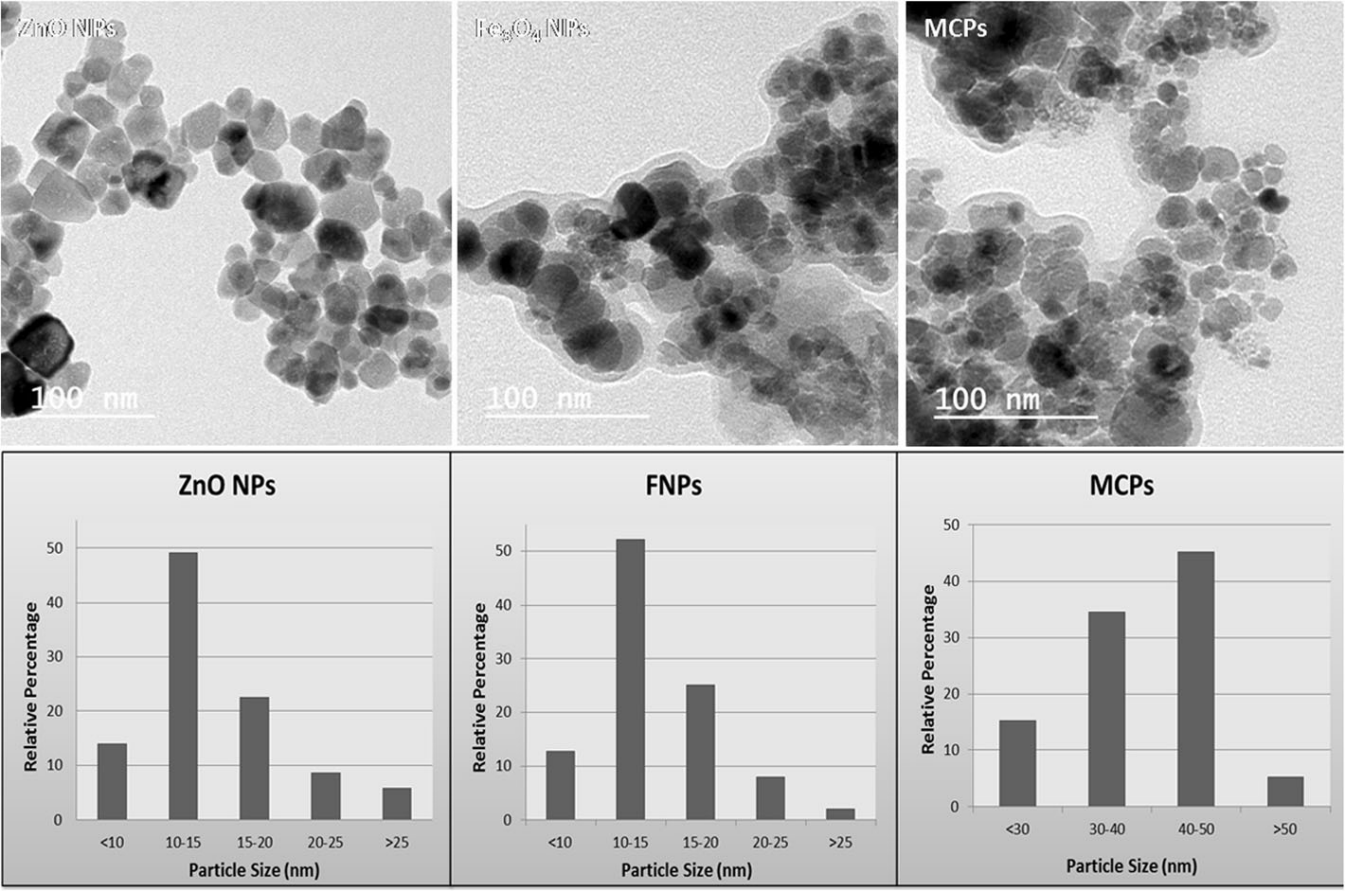
TEM images of ZnO NPs, FNPs (Fe3O4 NPs), and MCPs with histogram showing relative size distribution. This illustrates formation of composite on nanoscale as suggested by XRD spectra comprising both NPs

The particle size of MCPs was calculated to be 44.05 ± 1.2 nm from Scherrer’s equation with narrow distribution size. No any other peaks besides ZnO and Fe_3_O_4_ suggest that no impurities are present. TEM image in Fig. 2 shows morphological characteristic of particles which is consistent with XRD result being narrowly distributed and crystalline in nature. TEM image of MCPs shows increase in size to average 44 nm from core size of average 14 nm Fe_3_O_4_ NPs which clearly illustrates binding of ZnO NPs via trisodium citrate.

### FTIR Spectroscopy Analysis

**Fig. 3:**
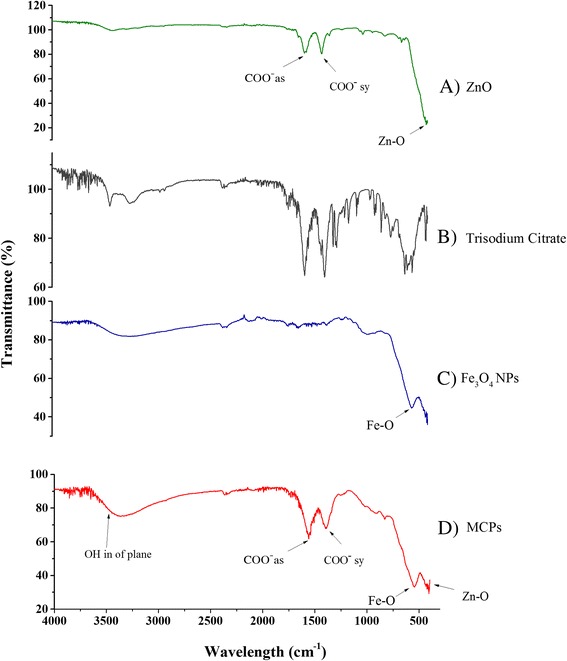
FTIR spectrum of *A* ZnO NPs, *B* trisodium citrate, *C* Fe_3_O_4_ NPs, and *D* magnetic composite NPs (MCPs) from 4000 to 300 cm^−1^. Characteristic peaks of metal oxide were observed at 410 and 545 cm^−1^ corresponding to vibrational mode of Zn–O and Fe–O bonds, respectively. Peaks corresponding to 1200–1600 cm^−1^ in ZnO NPs are of symmetric and asymmetric stretching of COO^−^ bond. These significant peaks of both ZnO and Fe_3_O_4_ NPs are also observed in MCPs signifying successful conjugation

Figure 3 shows FTIR spectrum of particles from 4000 to 400 cm^−1^. Characteristic peaks of metal oxide were observed at 410 and 545 cm^−1^ corresponding to vibrational mode of Zn–O and Fe–O bonds, respectively. These peaks are present in MCPs, as well as individually in ZnO and Fe_3_O_4_ nanoparticles. Peaks observed in between 1200 and 1600 cm^−1^ are due to symmetric and asymmetric stretching of COO^−^ bond which is observed to be sharper in case of asymmetric stretching of COO^−^, due to superposition of out of plane O–H vibration. O–H bond occurs due to physical adsorption of water on nanoparticle. These peaks corresponding to O–H and COO^−^ vibration along with characteristic peak for Zn–O and Fe–O impute that MCPs comprising of ZnO and Fe_3_O_4_ have formed after sodium citrate had been grafted on the surface of Fe_3_O_4_ due to the reaction between hydroxide groups on Fe_3_O_4_ and carboxylate anion of sodium citrate [18, 19]. Considerable decrease in FTIR peaks to that of trisodium citrate in MCPs further verifies that the sample has almost no impurities.

### VSM Analysis

**Fig. 4:**
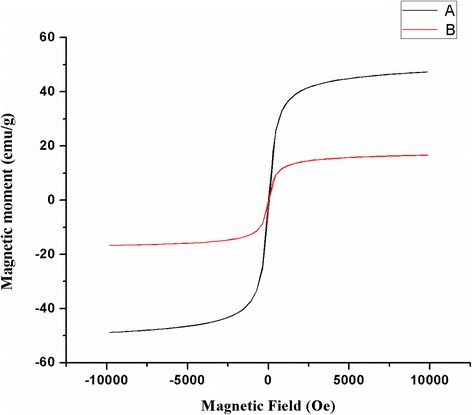
Magnetization curves measured at room temperature for *A* Fe_3_O_4_ NPs and *B* magnetic composite NPs (MCPs) between −10 and +10 kOe applied magnetic field. Obtained result shows that MCPs have retained superparamagnetic behavior with decrease in saturation magnetization

Figure 4 shows the magnetization curve of as-synthesized Fe_3_O_4_ NPs and MCPs versus applied magnetic field (H) between −10 and +10 kOe. For as-synthesized Fe_3_O_4_ NPs, the saturation magnetization (*M*_s_) is 47.22 emu/g which is reported to vary as a function of particle size and surface disorder [14, 20]. Similarly, the saturation magnetization of MCPs is 16.63 emu/g. Compared to Fe_3_O_4_ NPs, MCPs have lower *M*_s_ which is attributed to the presence of nonmagnetic ZnO shell on the surface of Fe_3_O_4_ through (i) interaction of surface atoms of magnetite with nonmagnetic layer of ZnO [20] and (ii) surface defects and strain in nanocomposite [21].

The remanence of as-synthesized Fe_3_O_4_ NPs and MCPs nanoparticles are 0.596 and 0.227 emu/g, respectively. Both possess negligible remanence at room temperature showing their typical superparamagnetic behavior. The coercivity of MCPs is obtained to be 62.90 Oe while that in as-synthesized Fe_3_O_4_ NPs is 64.17 Oe. This decrease in coercivity is attributed to decrease in inter-particle interactions and magnetoelastic anisotropy due to stress resulting from coating of magnetic core with ZnO shell [21]. All in all, MCPs have retained superparamagnetic behavior as compared to as-synthesized Fe_3_O_4_ NPs.

### Cytotoxicity Study

**Fig. 5:**
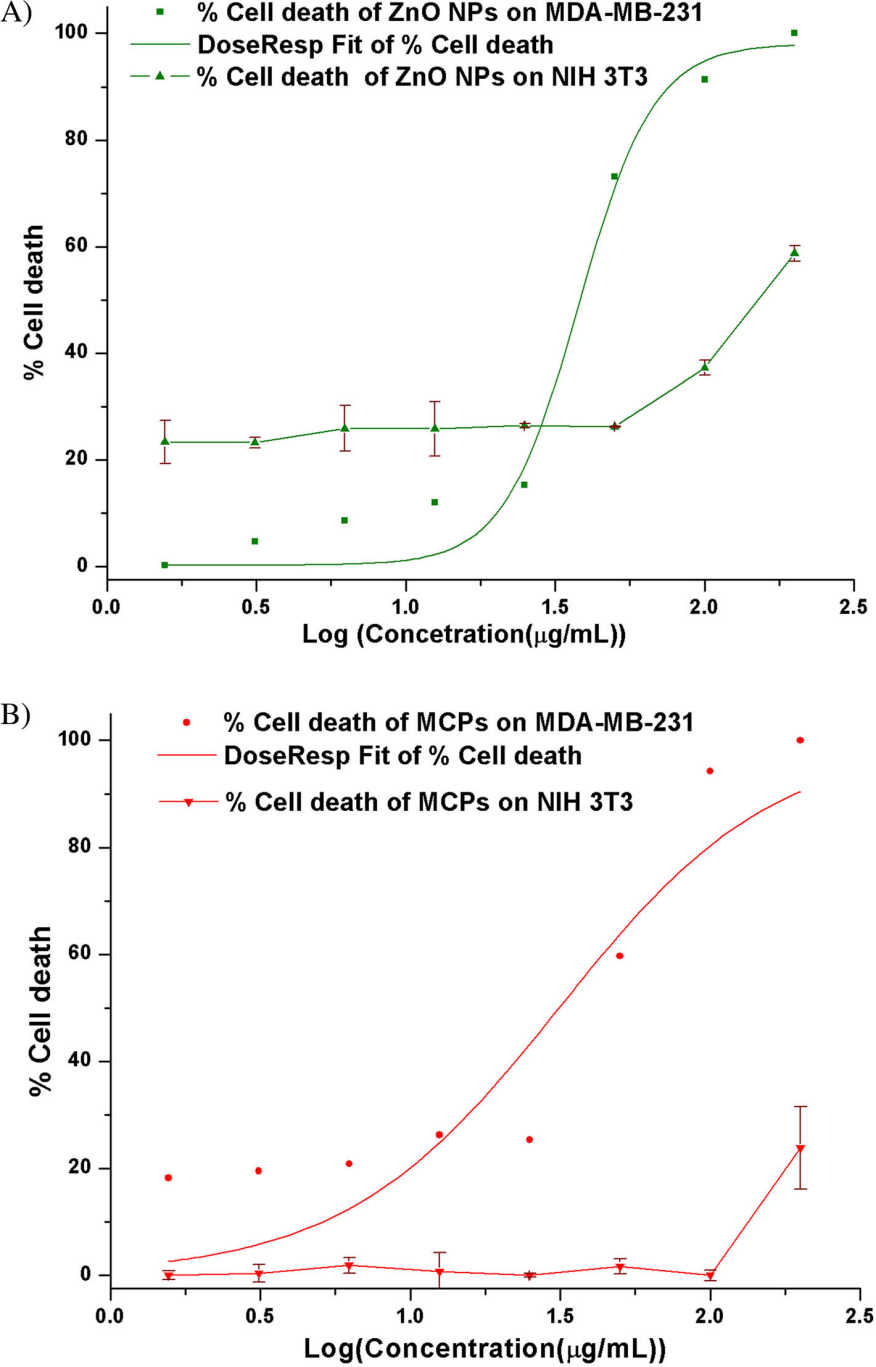
Dose-response curve of ZnO NPs (**a**) and MCPs (**b**) on cancer cell line MDA-MB 231 (Adj. *R* ^2^ = 0.91) and cytotoxicity plot ± SD of same agents on noncancerous cell NIH 3T3. Both agents show concentration independent and very less cytotoxicity on NIH 3T3 in comparison to dose-dependent strong response towards cancer cell (MDA-MB-231). This inherent selectivity of ZnO NPs, however, was found to be synergistically enhanced in MCPs with high potency (EC50 31.29 μg/mL for MCPs and 38.44 μg/mL for ZnO only) and significant hill slope (*P*) 1.21

Percent cell death computed from percent viability was plotted against logarithmic concentration of ZnO NPs and MCPs for MDA-MB-231 under inbuilt dose-response function of Origin 8. The fitted graph was shown in Fig. 5 with Adj. *R* ^2^ 0.91 along with percent cell death for NIH 3T3 cell line. EC50 value of ZnO NPs was found to be at 38.44 μg/mL whereas for MCPs, it was 31.29 μg/mL. This shows higher potency of MCPs compared to the ZnO NPs alone. Since only potency is not sufficient to describe efficiency of the drug, other parameters like hill slope and area under the curve were computed and compared to further illustrate advantage of the MCPs over naked ZnO NPs.

Hill slope (*p* value) for MCPs was 1.21 ± 0.26 and that of ZnO alone was 3.24 ± 1.05. Though the value is reduced, it is still positive and greater than 1 which signifies strong cooperativity between MCPs and cell death. This reduction may be particularly important for clinical application as steep dose-response might pose challenges in cases of low compliance and unfavorable pharmacokinetic profile [22].

The toxicity of ZnO NPs on NIH 3T3 significantly differs from that of MDA-MB-231 (*p* value < 0.05; two-factor ANOVA at *α* = 0.05). ZnO NPs show less toxicity on NIH 3T3 above concentration 50 μg/mL than that of MDA-MB-231 whereas below concentration 50 μg/mL, they retain certain toxicity. This, however, has been completely reduced in case of MCPs. Up to 100 μg/mL, the toxicity of MCPs on NIH 3T3 was found to be dose independent (Pearson’s *r* = −0.14) and very low. Given that selective killing of ZnO NPs between cancerous and noncancerous cells has been already quoted [7] and similar findings in our result also support this behavior [23], our synthesized MCPs show preferentiality between cancer and noncancerous cell. However, the further decrease in toxicity in NIH 3T3 (*p* value <0.05) in comparison to ZnO NPs elevate this effect. This may be because of less ZnO moieties on surface of MCPs compared to that of as-synthesized ZnO NPs alone or because of smaller particle size of ZnO facilitating more uptake initially showing certain toxicity to NIH 3T3. But, this should also affect cancer cell line in similar fashion which is seen in the decrement of hill slope in dose-response curve of MCPs.

**Fig. 6:**
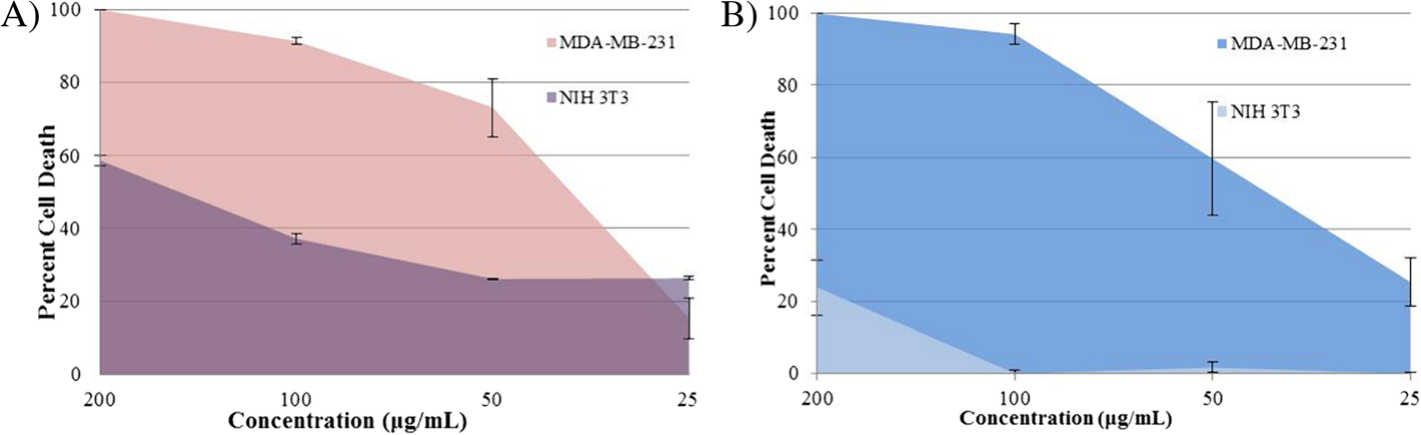
Plot of percent cell death ± SD against concentration (μg/mL) of **a** ZnO NPs and **b** MCPs on MDA-MB 231 and NIH 3T3 visually representing area under the curve (AUC) for measure of drug activity

Area under the relative viability curve (AUC), a parameter that combines efficacy and potency into single measure, was computed as a sum of measured responses (relative cell death) at all tested dose (concentrations) for ZnO NPs and MCPs on both cell lines [24] where higher AUC value corresponds to higher activity for cell death. AUC provides the robust measure for response when single drug is compared between various cell lines at the same concentration range. Difference in AUC for particular drug between cancer and normal cell is of particular importance for clinical purpose, and our results show that difference in AUC for MCPs between MDA-MB-231 and NIH-3T3 is 253.68 which is more than that of ZnO NPs, 131.45, for concentration range, 25 to 200 μg/mL as represented in Fig. 6. This greater difference in AUC, higher potency, and less toxicity towards normal cell make MCPs clinically significant compound for therapeutic use.

It should be noted that, from our XRD and FTIR results, the as-synthesized MCPs contain independent domain of ZnO and ferrite since no alloying or interaction between these to disturb their individual characteristic peaks have been seen. This imputes that the mechanism by which MCPs exert toxicity must be related with individual activity of NPs at given condition. Since researches in cytotoxicity of ZnO NPs show oxidative stress, ionic imbalance, and induction of apoptosis as major cause for their cytotoxicity [25], ROS imparting toxicity must be the probable route for our synthesized NPs. Ferrite as such is nontoxic [26], but in the presence of excess H_2_O_2_, Fe_3_O_4_ NPs can convert H_2_O_2_, a reactive oxygen species to more potent ROS and increase oxidative stress in cell [27]. So, it can be hypothesized that in case of MCPs, ZnO induces ROS including H_2_O_2_ and Fe_3_O_4_ NPs utilize these H_2_O_2_ as a substrate to produce more potent ROS triumphing over near-exhausted scavenging activity of cancer cell [28]. This explains the higher potency of MCPs and similar toxicity profile as of ZnO. The relative low abundance of ROS/H_2_O_2_ in normal cell compared to cancer cell [28, 29] and inherent nontoxic nature of ZnO NPs towards noncancerous cells cause MCPs to produce less ROS towards normal cell which can even be defended by antioxidant mechanism of cell itself and thus less toxicity, even 100% cell viability (0% cell death) as seen in Fig. 6.

It is important to note that this cytotoxic result is after 24 h of treatment and might not relate to chronic cytotoxic effect. The result of 100% cell viability of our MCPs in normal 3T3 cell, however, does not guarantee nontoxicity in the long run. But, nonetheless, the huge difference between cytotoxicity of our MCPs in cancer cell and noncancerous cell signifies the preferential cytotoxicity of as-synthesized MCPs.

### CV Staining Assay

**Fig. 7:**
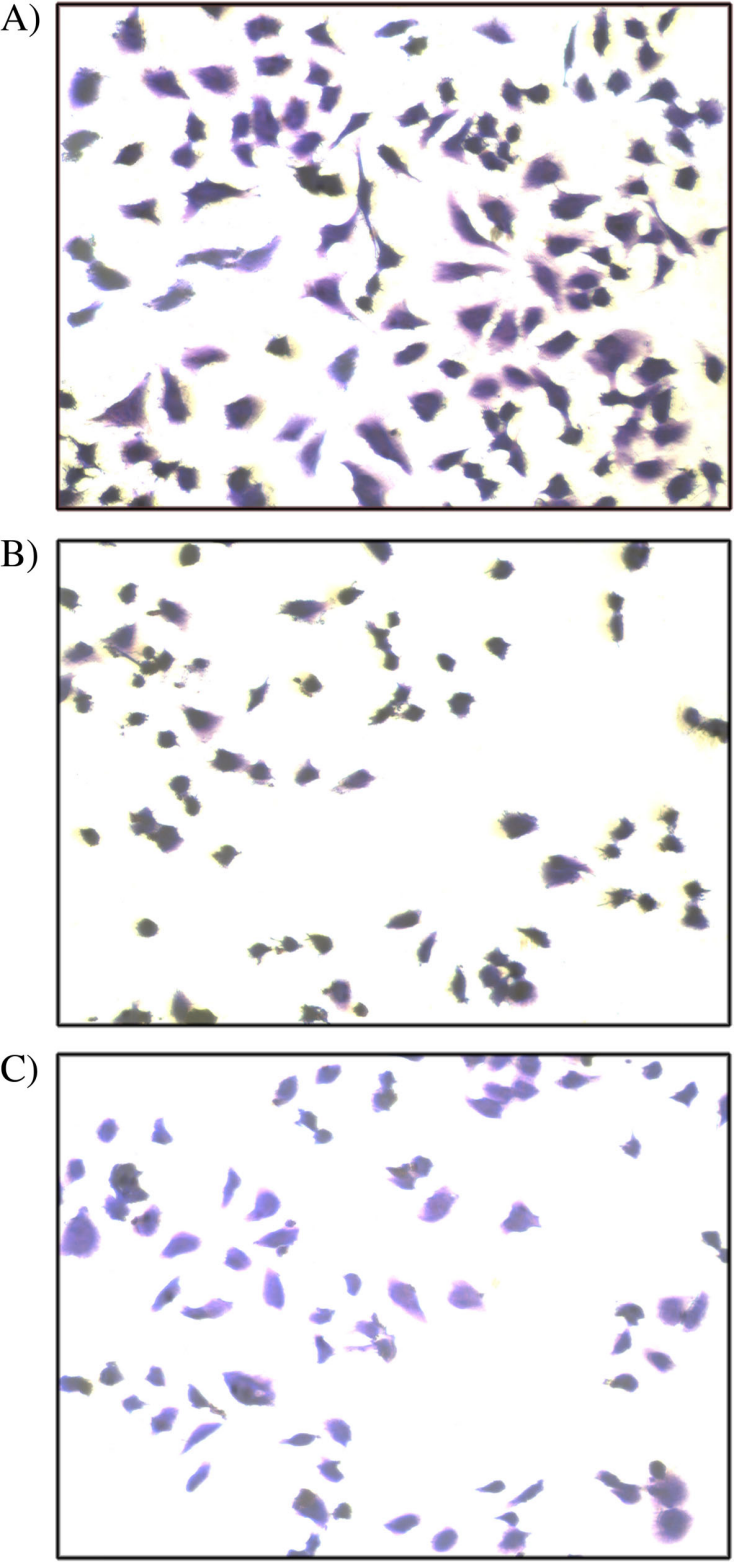
CV staining images of MDA-MB 231 cells. **a** Control (untreated) MDA-MB 231. **b** MCPs treated. **c** Naked ZnO NPs treated. Cells were treated with nanoparticles for 24 h at concentration of near EC50 value 30 and 40 μg/mL, respectively, for MCPs and ZnO NPs. Cells were fixed by 4% paraformaldehyde for 30 min and stained by 0.05%. Crystal violet for 30 min and then subsequently washed with distilled water followed by microscopic observation at ×10

Crystal violet (CV) staining was done to support the result of cytotoxicity depicted by MTT assay. Crystal violet dye (hexamethylpararosaniline) is a mixture of violet rosanilins that stains nucleus deep blue, aiding in their visualization [30]. Since nonadherent dead cells are removed during the washing process of this assay, cells observed as deep blue, stained by crystal violet, account for viable cells only. Result of CV staining, as depicted by Fig. 7, shows reduction of viable cells in case of MCP-treated MDA-MB-231 cells (B) compared to the untreated one (A) and, furthermore, is comparable to ZnO NP-treated cells (C). This observation is in accordance with the result of MTT assay corresponding to the cytotoxicity of MCPs at EC50 concentration on MDA-MB-231 and hence verifies the cytotoxicity of MCPs towards breast cancer cell.

## Conclusions

ZnO-Fe_3_O_4_ magnetic conjugated NPs retained inherent selective property of ZnO and magnetic property of Fe_3_O_4_ NPs and showed preferential cytotoxicity towards breast cancer cell line MDA-MB-231, with no significant cytotoxicity towards noncancerous NIH 3T3 cell. This finding suggests that MCPs, at a concentration range of 50–100 μg/ml, could be exploited for further research as a putative preferential anticancer agent. Also, the dramatic enhancement of selectivity shown by the conjugation of magnetic Fe_3_O_4_ NPs with ZnO NPs holds greater promises for effective clinical application. The magnetic property of MCPs can be used to target it to a particular site of cancerous tissue in our body using external magnetic field, while the selectivity property of MCPs can be used to selectively impart cytotoxicity in cancer cells of the targeted site, causing no significant harm to the normal cell around the site. Thus, ZnO-Fe_3_O_4_ MCPs can act as highly selective site-specific advanced NPs for targeted cancer therapy. However, further in-depth research on chronic toxicity profile of such composite including ROS production, antioxidant activity profiling, and study of electronic interaction of NPs is recommended for understanding detail mechanism behind this significant result and for possible in vivo application.

## Abbreviations

CV: Crystal violet
DMEM: Dulbecco’s modified Eagle’s medium
DPBS: Dulbecco’s phosphate buffer saline
Fe_3_O_4_ NPs: Iron (II III) oxide nanoparticles
FTIR: Fourier transform infrared
MCPs: Magnetic composite nanoparticles
MTT: Methyl thiazolyltetrazolium
ROS: Reactive oxygen species
TEM: Transmission electron microscopy
VSM: Vibrating sample magnetometry
XRD: X-ray diffraction
ZnO NPs: Zinc oxide nanoparticles
